# Significance of nanoparticles aggregation on the dynamics of rotating nanofluid subject to gyrotactic microorganisms, and Lorentz force

**DOI:** 10.1038/s41598-022-20485-0

**Published:** 2022-09-28

**Authors:** Bagh Ali, Imran Siddique, Rifaqat Ali, Jan Awrejcewicze, Fahd Jarad, Hamiden Abd El-Wahed Khalifa

**Affiliations:** 1 School of Mathematics and Statistics, Northwestern Polytechnical University, Xi’an, 710129 China; 2 Faculty of Sciences, Superior University, Lahore, 54000 Pakistan; 3 Department of Mathematics, University of Management and Technology, Lahore, 54770 Pakistan; 4 Department of Mathematics, College of Science and Arts, King Khalid University, Muhayil, Abha 61413 Saudi Arabia; 5 Department of Automation, Biomechanics and Mechatronics, Lodz University of Technology, 1/15 Stefanowskiego St., 90 924 Lodz, Poland; 6 Department of Mathematics, Cankaya University, Etimesgut, Ankara Turkey; 7 Department of Medical Research, China Medical University Hospital, China Medical University, Taichung, Taiwan; 8 Department of Mathematics, College of Sciences and Arts, Qassim University, Al-Badaya, 51951 Saudi Arabia; 9 Department of Operations Research, Faculty of Graduate Studies for Statistical Research, Cairo University, Giza, 12613 Egypt

**Keywords:** Mathematics and computing, Nanoscience and technology

## Abstract

The significance of nanoparticle aggregation, Lorentz and Coriolis forces on the dynamics of spinning silver nanofluid flow past a continuously stretched surface is prime significance in modern technology, material sciences, electronics, and heat exchangers. To improve nanoparticles stability, the gyrotactic microorganisms is consider to maintain the stability and avoid possible sedimentation. The goal of this report is to propose a model of nanoparticles aggregation characteristics, which is responsible to effectively state the nanofluid viscosity and thermal conductivity. The implementation of the similarity transforQ1m to a mathematical model relying on normal conservation principles yields a related set of partial differential equations. A well-known computational scheme the FEM is employed to resolve the partial equations implemented in MATLAB. It is seen that when the effect of nanoparticles aggregation is considered, the temperature distribution is enhanced because of aggregation, but the magnitude of velocities is lower. Thus, showing the significance impact of aggregates as well as demonstrating themselves as helpful theoretical tool in future bioengineering and industrial applications.

## Introduction

Nanofluids are made by suspending nanoparticles in a liquid carrier such as oil, argon, or ethylene glycol^1^. The presence of nanomaterials in the host fluid has a significant impact on the thermophysical features of base fluids with low conductivity properties, according to theoretical and experimental findings^2–4^. Due to their interesting uses in every aspect of science and engineering, the convective nanofluid thermal transport flow attention a large number of researchers. To mention several, the ceramic nanomaterials and diamond are utilized to improve the mineral-oil dielectric properties, the liquid incorporated nanomaterials can be utilized for directly sunlight absorption in solar collectors, making them suitable for biomedical uses including cancer therapy and drug delivery etc.^5–7^. The several numerically computational have been studied to enhance the fluid thermal conductivity like, peristaltic pumping of a nanofluid^8^, Casson fluid incorporated nanoparticles^9^, magnetized nanoparticles subject to water as a host fluid^10^, hybrid nanoparticles considered to enhance the performance of DC operated micropump^11^, non-uniform heat source/sink with nanoparticles incorporated in the base fluid to observe the heat transfer rate^12^, thermal enhancement through multi-twisted tape subject to tiny particles^13^, and hydrothermal nanofluid analysis subject to wavy pipe geometry^14^.

The rotatory flow has wide range of applications in real life, such as turbine rotors, air cleaner devices, mixing materials machinery, medical field, and power generation systems, etc.^15,16^. The first endeavor towards the rotating path of fluid was made by Wang^17^. Many researchers are investigated the rotating flow under different aspects and geometries are given in Refs.^18–21^. The presence of a density gradient in the flow field causes the bio convective phenomenon. Consequently, the movement of the particles at the macroscopic level causes the improvement of the density stratification of the base liquid in one direction. Many researchers were interested in the existence of such Gyrotactic microorganisms in the nanofluid flow because of their potential applications in enzymes, biotechnology, biosensors, biofuels, and medication delivery. These applications prompted a number of investigators to do numerical simulations on bio convective nanofluid flow with microorganisms passing through a variety of flow fields. Chu et al.^22^ have used Homotopy Analysis Approach to evaluate numerically bio convection Maxwell nanofluid flow via bidirectional periodically moving plate under nonlinear radiation and heat source phenomena. Rao et al.^23^ scrutinized the bio convective flow in a conventional reactive nanofluid towards the isothermal upright cone with Gyrotactic microorganisms immersed in a permeable medium. Awais et al.^24^ investigated assisting and opposing bio convective nanofluid flow with motile microorganisms numerically via Adams–Bash forth approach (ABA). Abdelmalek et al.^25^ investigated bio-convective third-grade nanofluid stream over an extending sheet under Arrhenius activation energy by using bvp4c. Shafiq et al.^26^ investigated the chemically reactant bio-convective second grade nanofluid flow under buoyancy effect.

Numerous investigators came to the conclusion that particle aggregation^27,28^, particle motion^29^ and liquid-layering^30^ are most valuable variables in thermal conductivity processes in nanofluids. The fact that particle aggregation can improve nanofluids’ efficient thermal conductivity has been demonstrated experimentally^30,31^. According to Wang et al.^32^, particle clustering could have a noteworthy effect on the improvement of thermal conductivity of nanoliquid. In^33^, authors proposed a mixture model to describe two-component heterogeneous structures. The particle aggregation form is invariable in their model that ignores the impact of aggregation shape on nanofluids effective thermal conductivity.

The extensive literature review stated above reveals that the minimal attention to the self-motile thermophile microorganisms ingrained nanofluid rotating flow across a stretching sheet with the impact of the external magnetic field subject to nanoparticles aggregation. According to the author’s insight, none of the listed articles discuss the detailed problem. The main objective of this study is to examine the heat and mass transport impacts of transitory hydromagnetic rotating nanofluid three-dimensional flows with Gyrotactic microbes. Numerous scholars have lately examined the hydromagnetics nanofluid flow for Newtonian and non-Newtonian flow^34–36^ by utilizing variational finite element technique. The coupled non-linear PDEs is resolved using a control volume technique with a weighted residual approach using a Galerkin FEM^37,38^. The flow field characteristics for a variety of important parameter modifications are explored and illustrated graphically. The MATLAB code blocks yielded computational findings that were validated by existing literature and determined to have a reasonable correlation. This numerical analysis applies to gasoline, polymers, nutrition release precision, engine lubricants, paint rheology, Bio-Sensors, medicine delivery, and biofuels.

### Research questions

The following relevant scientific research questions are examined in the study:

1. To explore the impact of Coriolis force and Lorentz force on thermal, momentum, and concentration profiles in the presence and absence of nanoparticle aggregation?
2. What impact do the Coriolis and Lorentz forces have on mass transport rate, skin friction factor, and thermal efficiency presence and absence of nanoparticle aggregation?
3. What are the impacts of Brownian motion, thermophoresis, and time-dependent parameters on thermal distribution?
4. Evaluate how bio-convection affects the microorganisms profile in the presence and absence of nanoparticle aggregation?

## Mathematical formulation

**Figure 1:**
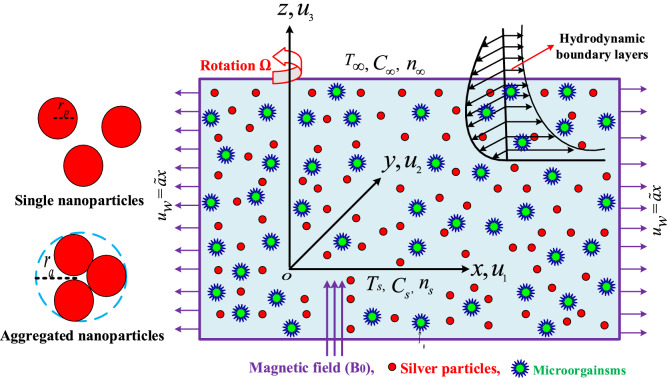
Physical representation of problem.

Consider a MHD three-dimensional rotating Maxwell nanofluid flow across a bidirectional stretching surface. Figure 1 depicts the fluid dynamic structure and three-dimensional the developed problem. The flow is limited to $z\ge 0$. The fixed origin *O*(*x*, *y*, *z*) has been chosen, with the *x*-*axis* depicting the stretching surface’s movement, the y axis depicting the surface’s normal, and the *z*-*axis* depicting transverse to the *xy* plane. A static and uniform magnetic *B*0 field is applied in the axial direction (z-direction). Due to the low magnetic Reynolds number, a reduced magnetic field is created, hence Ohmic dissipation and Hall current are negligible^39^. $T_\infty$, $N_w$, $C_\infty$, represents ambient temperature and concentration and $T_w$, $N_w$, $C_w$, signifies surface temperature and concentration. To avoid sedimentation, gyrotactic microorganisms is taken into account to maintain convection stability. $V = (u_1(x,y,z), u_2(x,y,z), u_3(x,y,z))$ considers the velocity field in the current complicated situation. The physical properties of nanoparticles aggregation and without aggregation, and based fluid are mentioned in the Tables 1 and 2. The governing equations of continuity, momentum, temperature, concentration and bioconvection of the fluid flow are given as^40–42^:

1 $$\begin{aligned}{}&{u}_{1x}+{u}_{2y}+{u}_{3z}=0, \end{aligned}$$

2 $$\begin{aligned}{}&\rho _{nf}({u}_{1t}+ {u}_1{u}_{1x} + {u}_2{u}_{1y} + {u}_3{u}_{1z} + 2\Omega {u}_2)= \mu _{nf}{u}_{1zz} - \sigma _{n_f}B_0^2u_1, \end{aligned}$$

3 $$\begin{aligned}{}&\rho _{nf}({u}_{2t}+ {u}_1{u}_{2x} + {u}_2{u}_{2y} + {u}_3{u}_{2z} - 2\Omega {u}_1)= \mu _{nf}{u}_{2zz}- \sigma _{n_f}B_0^2u_2, \end{aligned}$$

4 $$\begin{aligned}{}&{T}_t + {u}_1{T}_x + {u}_2{T}_y +{u}_3{T}_z = \alpha _{n_f}{T}_{zz} +{\tau }^*\{D_b{C}_z{T}_z +\frac{D_T}{T_\infty }{T}_z^2\}, \end{aligned}$$

5 $$\begin{aligned}{}&{C}_t + {u}_1{C}_x + {u}_2{C}_y +{u}_3{C}_z = {D}_b{C}_{zz} +\frac{D_T}{ T_\infty }{T}_{zz}, \end{aligned}$$

6 $$\begin{aligned}{}&{n}_t + {u}_1{n}_x + {u}_2{n}_y +{u}_3{n}_z + \frac{bWc}{(C_s-C_\infty )}\left[ (n{C}_z)_z\right] = D_m{N}_{zz}, \end{aligned}$$

where $\rho _{nf}, \mu _{n_f}, \alpha _{n_f}$, are the fluid density, dynamic viscosity and thermal diffusivity, *C* indicates the nanoparticles concentration, *n* symbolizes microorganisms concentration, *T* represnts the fluid temperature, $D_{T}$, $D_{N}$, and $D_{B}$, are represents the thermophoretic diffusion coefficient, diffusivity of microorganisms, and Brownian diffusion coefficients, respectively. The boundary constraints are^43,44^:

7 $$\begin{aligned}{}&t <0: {u}_1 = 0,\ {u}_2 = 0,\ {u}_3 = 0,\ {C} = {C}_\infty ,\ {T} = {T}_\infty ,\ {n} = {n}_\infty , \end{aligned}$$

8 $$\begin{aligned}{}&t\ge 0: {u}_1 =\tilde{a}x,\ {u}_3 = {u}_2 =0,\ {T} = {T}_s,\ {C} = {C}_s,\ {n} = {n}_s,\ as\ z=0, \end{aligned}$$

9 $$\begin{aligned}{}&t\ge 0: {u}_1\rightarrow 0,\ {u}_2\rightarrow 0, \ {T}\rightarrow {T}_\infty ,\ {C}\rightarrow {C}_\infty , \ {n}\rightarrow {n}_\infty ,\ as\ z \rightarrow \infty . \end{aligned}$$

Similarity transformations (see^40,43^):

10 $$\begin{aligned} \left. \begin{aligned} u_1= \tilde{a}x\frac{\partial F_1(\Gamma ,\eta )}{\partial \eta },\ u_2 = \tilde{a}xF_2(\Gamma ,\eta ),\ u_3 = -\sqrt{\tilde{a}\nu \Gamma }F_1(\Gamma ,\eta ),\ \ \Gamma = 1-e^{-\zeta }, \eta = \sqrt{\frac{\tilde{a}xz^2}{\Gamma \nu }},\ \\ \zeta = \tilde{a}t,\ \frac{T-T_\infty }{(T_s-T_\infty )} = \Theta (\Gamma ,\eta ), \ \frac{C-C_\infty }{(C_s-C_\infty )}= \Phi (\Gamma ,\eta ),\ \frac{n-n_\infty }{(n_s-n_\infty )} = \chi (\Gamma ,\eta ). \end{aligned}\right\} \end{aligned}$$

**Table 1:** Thermo-physical properties of water base fluid and nanoparticles^45^.

Physical properties	$$\rho$$ (kg m$$^{-3})$$	$$C_p$$ (J/kg K)	$$\kappa$$ (W/m K)
$\hbox {H}_2\hbox {O}$	0991.1	4179.0	00.613
$\hbox {TiO}_2$	4250.0	686.20	8.9538

**Table 2:** Thermo-physical attributes of base fluid and nanoparticles^45,46^.

Properties	With aggregation	Without aggregation
viscosity $(\mu )$	$\frac{\mu _{n_f}}{\mu _{f}}={(1-\frac{\Phi _{ag}}{\Phi _{m}})^{2.5\Phi _m}}$	$\frac{\mu _{n_f}}{\mu _{f}}=\frac{1}{(1-\Phi )^{2.5}}$
density $(\rho )$	$\rho _{n_f}=\rho _f(1-\Phi _{ag})+\Phi _{ag}\rho _s$	$\rho _{n_f}=\rho _f(1-\Phi )+\Phi \rho _s$
Heat capacity$(\rho C_p)$	$(\rho C_p)_{nf}=(\rho C_p)_f(1-\Phi _{ag})+\Phi _{ag}\frac{(\rho C_p)_s}{(\rho C_p)_f}$	$(\rho C_p)_{nf}=(\rho C_p)_f(1-\Phi )+\Phi \frac{(\rho C_p)_s}{(\rho C_p)_f}$
Thermal conductivity($\kappa$)	$\frac{k_{n_f}}{k_f}=\frac{k_{ag}+2k_f-2\Phi _{ag}(k_f-k_{ag})}{k_{ag}+2k_f + \Phi _{ag}(k_f-k_{ag})}$	$\frac{k_{n_f}}{k_f}=\frac{k_s+2k_f-2\Phi (k_f-k_s)}{k_s+2k_f + \Phi (k_f-k_s)}$

In view of Eq. (10), Eq. (1) is satisfied and Eqs. (2–9) becomes non-linear PDEs into transformed coordinate systems ($\Gamma ,\eta$).

11 $$\begin{aligned}{}&\frac{1}{\chi _1\chi _2}F_1'''+ 0.5\eta {F_1''}-0.5\Gamma \eta {F_1''}+\Gamma (F_1F_1''-F_1'^{2} - \frac{M^2}{\chi _2}F_1'+ 2\lambda {F_2}) -\Gamma (1-\Gamma )\frac{\partial {F_1}'}{\partial \Gamma }=0, \end{aligned}$$

12 $$\begin{aligned}{}&\frac{1}{\chi _1\chi _2}F_2''+ 0.5\eta {F_2'}-0.5\Gamma \eta {F_2'}+\Gamma (F_1F_2'- 2\lambda {F_1}'- \frac{M^2}{\chi _2}F_2-F_1'F_2)-\Gamma (1-\Gamma )\frac{\partial {F_2}}{\partial \Gamma }=0, \end{aligned}$$

13 $$\begin{aligned}{}&\frac{\chi _3}{\chi _4}\Theta '' + 0.5\eta (1-\Gamma )P_r\Theta ' +\Gamma {P_r}F_1\Theta ' +N_bP_r\Theta \Phi +N_tP_r\Theta '^2- \Gamma (1-\Gamma )P_r\frac{\partial \Theta }{\partial \Gamma }=0, \end{aligned}$$

14 $$\begin{aligned}{}&\Phi '' + 0.5\eta {S_c}(1-\Gamma )\Phi '+S_c\Gamma {F_1}\Phi ' + N_t{N^{-1}_b}\Theta '' - \Gamma (1-\Gamma )S_c\frac{\partial \Phi }{\partial \Gamma }=0, \end{aligned}$$

15 $$\begin{aligned}{}&\chi '' + \frac{{S_b}}{2}(1-\Gamma )S_b\chi '+\Gamma {S_b}{F_1}\chi ' -P_e\Phi ''(\delta _1+\chi )+P_e\chi '\Phi ' = S_b\Gamma (1-\Gamma )\frac{\partial \chi }{\partial \Gamma }, \end{aligned}$$

16 $$\begin{aligned}{}&\left. \begin{aligned} \lim _{\eta \rightarrow 0}F_1(\Gamma ,\eta )= 0,\ \lim _{\eta \rightarrow 0}F_1'(\Gamma ,\eta ) =1, \lim _{\eta \rightarrow 0}F_2(\Gamma ,\eta ) = 0, \lim _{\eta \rightarrow 0} \Theta (\Gamma ,\eta ) = \lim _{\eta \rightarrow 0} \Phi (\Gamma ,\eta ) = \lim _{\eta \rightarrow 0} \chi (\Gamma ,\eta ) = 1,\ \Gamma \ge = 0,\\ \lim _{\eta \rightarrow \infty }F_1'(\Gamma ,\eta )\rightarrow 0, \lim _{\eta \rightarrow \infty } F_2(\Gamma ,\eta )\rightarrow 0, \lim _{\eta \rightarrow \infty } \Theta (\Gamma ,\eta )\rightarrow 0, \lim _{\eta \rightarrow \infty }\Phi (\Gamma ,\eta )\rightarrow 0, \lim _{\eta \rightarrow \infty } \chi (\Gamma ,\eta )\rightarrow 0,\ \Gamma \ge 0, \end{aligned}\right\} \end{aligned}$$

where

$$\begin{aligned} \chi _1&= {\left( 1-\frac{\Phi _{ag}}{\Phi _{m}}\right) ^{-2.5\Phi _m}},\ \chi _2 = (1-\Phi _{ag})+\Phi _{ag}\frac{\rho _{ag}}{\rho _f},\ \chi _3 = \frac{k_{ag}+2k_f-2\Phi _{ag}(k_f-k_{ag})}{k_{ag}+2k_f + \Phi _{ag}(k_f-k_{ag})},\\ \chi _4&= (1-\Phi _{ag})+\Phi _{ag}\frac{(\rho {C_p})_{ag}}{(\rho {C_p})_f}, \end{aligned}$$

and $\lambda = \frac{\Omega }{{a}}$ signifies rotating parameter, $M = \sqrt{\frac{\sigma _{n_f}B_o^2}{\rho _f\tilde{a}}}$ deliberated the magnetic parameter, $P_r = \frac{\nu }{\alpha _{n_f}}$ symbolize the Prandtl number, $S_c= \frac{\nu }{{D}_b}$ is the Schmidt number $S_b = \frac{\nu }{{D}_m}$ represent bioconvection Schmidt number , $N_b = \tau \nu ^{-1}{D}_B ({C}_s -{C}_\infty )$ is the Brownian motion, $N_t = \frac{{D}_T (\tau {T}_s-\tau {T}_\infty )}{\nu {T}_\infty }$ represent the thermophoresis , $P_e = \frac{bW_c}{{D}_m}$ Peclet number, $\delta _1 = \frac{{n}_\infty }{{n}_s-{n}_\infty }$ is microorganism-concentration difference.

The following are the local skin friction coefficients, Sherwood coefficients, and Nusselt coefficients respectively as follows:

17 $$\begin{aligned} Nu= & {} \frac{xq_w}{\kappa ({T}_s-{T}_\infty )}, \end{aligned}$$

18 $$\begin{aligned} Shr= & {} \frac{xq_m}{{D}_b({C}_s-{C}_\infty )}, \end{aligned}$$

19 $$\begin{aligned} C_{f_x}= & {} \frac{\tau _w^x}{\rho {u}_1^2} , \end{aligned}$$

20 $$\begin{aligned} C_{f_y}= & {} \frac{\tau _w^y}{\rho {u}_1^2}. \end{aligned}$$

Using Eq. (10), we derive the following results:

21 $$\begin{aligned} \left\{ \begin{aligned}& C_{f_x}{Re_x}^{1/2} = \frac{{F_1}''(0)}{\sqrt{\Gamma }}, C_{f_y}{Re_x}^{1/2} = \frac{{F_2}'(0)}{\sqrt{\Gamma }},\\ & Nu_x{Re_x}^{1/2} = -\frac{\left[ \Theta '(0)\right] }{\sqrt{\Gamma }}, Shr_x{Re_x}^{1/2} = - \frac{\left[ \Phi '(0)\right] }{\sqrt{\Gamma }}. \end{aligned} \right. \end{aligned}$$

## Numerical procedure

The FEM is renowned for its ability to solve several types of DE. This process utilizes continuous piecewise approximation to reduce the amount of the inaccuracy^47^. The critical phases and a wonderful depiction of this method are laid out by Reddy^48^ and jyothi^49^. Because to its precision and computability, experts believe this numerical approach is a particularly effective instrument for solving current engineering and industrial challenges^50,51^. To solve Eq. (11) to (15) together with boundary condition (18), take this into consideration:

22 $$\begin{aligned} F_1'= H, \end{aligned}$$

Equations (11)–(16) are simplified to a lower order:

23 $$\begin{aligned}{}&\frac{1}{\chi _1\chi _2}H''+0.5(1-\Gamma )\eta {H}'+\Gamma ({F_1}{H}'-{H}^2+2\lambda {F_2}-\frac{M^2}{\chi _2}H) = \Gamma (1-\Gamma )\frac{\partial {H}}{\partial \Gamma }, \end{aligned}$$

24 $$\begin{aligned}{}&\frac{1}{\chi _1\chi _2}F_2''+\frac{1}{2}(1-\Gamma )\eta {F_2}'+\Gamma ({F_1}{F_2}'-{H}{F_2}-2\lambda {H}-\frac{M^2}{\chi _2}F_2) = \Gamma (1-\Gamma )\frac{\partial {F_2}}{\partial \Gamma }, \end{aligned}$$

25 $$\begin{aligned}{}&\frac{\chi _3}{\chi _4}\Theta ''+ 0.5\eta (1-\Gamma )P_r\Theta '+P_r\Gamma {F_1}\Theta '+ N_bP_r\Theta '\Phi ' + N_tP_r\Theta '^2 = P_r\Gamma (1-\Gamma )\frac{\partial {\Theta }}{\partial \Gamma }, \end{aligned}$$

26 $$\begin{aligned}{}&\Phi ''+ 0.5S_c(1-\Gamma )\eta \Phi '+S_c\Gamma {F_1}\Phi '+ {N_t}{N^{-1}_b}\Theta ''^2 = \Gamma (1-\Gamma )S_c\frac{\partial {\Phi }}{\partial \Gamma }, \end{aligned}$$

27 $$\begin{aligned}{}&\chi '' + \frac{{S_b}}{2}(1-\Gamma )\eta \chi '+\Gamma {S_b}{F_1}\chi ' -P_e\Phi ''(\delta _1+\chi )+P_e\chi '\Phi ' = S_b\Gamma (1-\Gamma )\frac{\partial \chi }{\partial \Gamma }, \end{aligned}$$

28 $$\begin{aligned}{}&\left. \begin{aligned} \lim _{\eta \rightarrow 0}F_1(\Gamma ,\eta )=0,\ \lim _{\eta \rightarrow 0}H(\Gamma ,\eta )=1, \lim _{\eta \rightarrow 0} F_2(\Gamma ,\eta )=0,\ \lim _{\eta \rightarrow 0}\Theta (\Gamma ,\eta )=\lim _{\eta \rightarrow 0}\Phi (\Gamma ,\eta )=\lim _{\eta \rightarrow 0}\chi (\Gamma ,\eta ) = 1,\ \Gamma \ge = 0,\\ \lim _{\eta \rightarrow \infty }H(\Gamma ,\eta )\rightarrow 0,\ \lim _{\eta \rightarrow \infty } F_2(\Gamma ,\eta )\rightarrow 0,\ \lim _{\eta \rightarrow \infty } \Theta (\Gamma ,\eta )\rightarrow 0,\ \lim _{\eta \rightarrow \infty } \Phi (\Gamma ,\eta )\rightarrow 0,\ \lim _{\eta \rightarrow \infty } \chi (\Gamma ,\eta )\rightarrow 0,\ \Gamma \ge 0. \end{aligned}\right\} \end{aligned}$$

The plate thickness $\eta =6.0$ and length $\Gamma =1.0$ are fixed for numerical computations. Equations (22)–(27) have a variational form that may be represented as:

29 $$\begin{aligned}{}&\int \limits _{\Omega _e}w_{f_1}\{F_1'-H\}d\Omega _e = 0, \end{aligned}$$

30 $$\begin{aligned}{}&\int \limits _{\Omega _e}w_{f_2}\bigg \{\frac{1}{\chi _1\chi _2}H''+\frac{1}{2}(1-\Gamma )\eta {H}'+\Gamma ({F_1}{H}'-{H}^2+2\lambda {H}-M^2H)- \Gamma (1-\Gamma )\frac{\partial {H}}{\partial \Gamma }\bigg \}d\Omega _e= 0, \end{aligned}$$

31 $$\begin{aligned}{}&\int \limits _{\Omega _e}w_{f_3}\bigg \{\frac{1}{\chi _1\chi _2}F_2''+\frac{1}{2}(1-\Gamma )\eta {F_2}'+\Gamma ({F_1}{F_2}'-{H}{F_2}-2\lambda {H})- \Gamma (1-\Gamma )\frac{\partial {F_2}}{\partial \Gamma }\bigg \}d\Omega _e = 0, \end{aligned}$$

32 $$\begin{aligned}{}&\int \limits _{\Omega _e}w_{f_4}\bigg \{\frac{\chi _3}{\chi _4}\Theta ''+ \frac{P_r}{2}(1-\Gamma )\eta \Theta '+P_r\Gamma {F_1}\Theta '+ N_bP_r\Theta '\Phi ' + N_tP_r(\Theta ')^2 - P_r\Gamma (1-\gamma )\frac{\partial \Theta }{\partial \Gamma }\bigg \}d\Omega _e = 0, \end{aligned}$$

33 $$\begin{aligned}{}&\int \limits _{\Omega _e}w_{f_5}\bigg \{\Phi ''+ 0.5S_c\eta (1-\Gamma )\Phi '+\Gamma {S_cF_1}\Phi '+ \frac{N_t}{N_b}(\Theta '')^2 -\Gamma (1-\Gamma )S_c\frac{\partial \Phi }{\partial \Gamma }\bigg \}d\Omega _e = 0, \end{aligned}$$

34 $$\begin{aligned}{}&\int \limits _{\Omega _e}w_{f_6}\bigg \{\chi '' + \frac{{S_b}}{2}(1-\Gamma )\eta \chi '+\Gamma {S_b}{F_1}\chi ' -P_e\bigg (\Phi ''(\delta _1+\chi )+\chi '\Phi '\bigg )- \Gamma (1-\Gamma )S_b\frac{\partial \chi }{\partial \Gamma }\bigg \}d\Omega _e= 0. \end{aligned}$$

**Figure 2:**
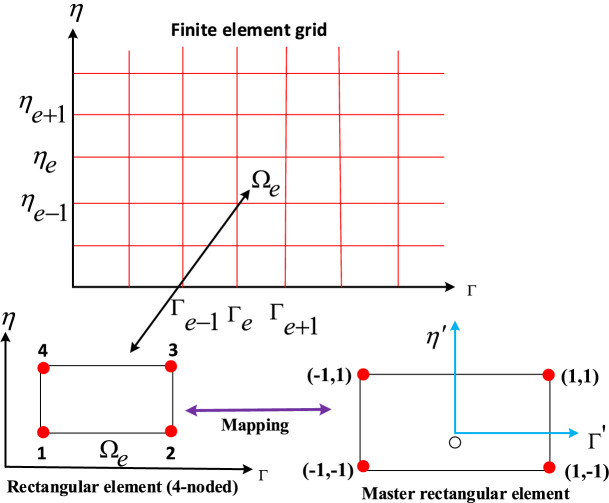
Finite element mesh and grid.

Here $w_{f_s} (s = 1,2,3,4,5,6)$ indicates the trial functions. Let divide the input ($\Omega _e$) split into four nodded components (see Fig. 2). The following are finite element estimations:

35 $$\begin{aligned} F_1 = \sum _{j=1}^4 F_{1j} \Upsilon _j(\gamma ,\eta ),\ H = \sum _{j=1}^4 H_j \Upsilon _j(\Gamma ,\eta ),\ F_2 = \sum _{j=1}^4 F_{2j} \Upsilon _j(\Gamma ,\eta ),\ \Theta = \sum _{j=1}^4 \Theta _j \Upsilon _j(\Gamma ,\eta ),\ \Phi = \sum _{j=1}^4\Phi _j\Upsilon _j(\Gamma ,\eta ). \end{aligned}$$

Here, $\Upsilon _j$ (j = 1,2,3,4) are the linear interpolation shapes functions for $\Omega _e$ as:

36 $$\begin{aligned} \left. \begin{aligned} \Upsilon _1 = \frac{(\Gamma _{e+1}-\Gamma )(\eta _{e+1}-\eta )}{(\Gamma _{e+1}-\Gamma _e)(\eta _{e+1}-\eta _e)},\ \Upsilon _2 = \frac{(\Gamma -\Gamma _e)(\eta _{e+1}-\eta )}{(\Gamma _{e+1}-\Gamma _e)(\eta _{e+1}-\eta _e)},\\ \Upsilon _3 = \frac{(\Gamma -\Gamma _e)(\eta -\eta _e)}{(\Gamma _{e+1}-\Gamma _e)(\eta _{e+1}-\eta _e)},\ \Upsilon _4 = \frac{(\Gamma _{e+1}-\Gamma )(\eta -\eta _e)}{(\Gamma _{e+1}-\Gamma _e)(\eta _{e+1}-\eta _e)}. \end{aligned}\right\} \end{aligned}$$

The following is the developed finite element model of the equations:

37 $$\begin{aligned} \begin{bmatrix} [L^{11}] &{} [L^{12}] &{} [L^{13}] &{} [L^{14}] &{} [L^{15}] &{} [L^{16}]\\ [L^{21}] &{} [L^{22}] &{} [L^{23}] &{} [L^{24}] &{} [L^{25}] &{} [L^{26}]\\ [L^{31}] &{} [L^{32}] &{} [L^{33}] &{}[ L^{34}] &{} [L^{35}] &{} [L^{36}]\\ [L^{41}] &{} [L^{42}] &{} [L^{43}] &{} [L^{44}] &{} [L^{45}] &{} [L^{45}]\\ [L^{51}] &{} [L^{52}] &{} [L^{53}] &{} [L^{54}] &{} [L^{55}] &{} [L^{56}]\\ [L^{61}] &{} [L^{62}] &{} [L^{63}] &{} [L^{64}] &{} [L^{65}] &{} [L^{66}] \end{bmatrix} \begin{bmatrix} \{F_1\}\\ \{H\}\\ \{F_2\} \\ \{\Theta \}\\ \{\Phi \}\\ \{\chi \} \end{bmatrix} = \begin{bmatrix} \{R_1\} \\ \{R_2\}\\ \{R_3\} \\ \{R_4\}\\ \{R_5\}\\ \{R_6\} \end{bmatrix} \end{aligned}$$

where $[L_{mn}]$ and $[R_m]$ (m, n = 1, 2, 3, 4) matrices are written as:

$$\begin{aligned} L^{11}_{ij}= & {} \int \limits _{\Omega _e} \Upsilon _i\frac{d\Upsilon _j}{d\eta }d\Omega _e, L^{12}_{ij} = -\int \limits _{\Omega _e} \Upsilon _i\Upsilon _jd\Omega _e, L^{13}_{ij} = L^{14}_{ij} = L^{15}_{ij} = L^{21}_{ij} = L^{24}_{ij} = L^{25}_{ij} = L^{26}_{ij} = 0, \\ L^{22}_{ij}= & {} -\frac{1}{\chi _1\chi _2}\int \limits _{\Omega _e} \frac{d\Upsilon _i}{d\eta } \frac{d\Upsilon _j}{d\eta }d\Omega _e +\frac{1}{2}(1-\Gamma )\eta \int \limits _{\Omega _e}\Upsilon _i \frac{d\Upsilon _j}{d\eta }d\Omega _e+\Gamma \int \limits _{\Omega _e} \bar{F_1}\Upsilon _i\frac{d\Upsilon _j}{d\eta }d\Omega _e-\Gamma \int \limits _{\Omega _e}\bar{H}\Upsilon _i \Upsilon _j d\Omega _e\\&- \frac{M^2}{\chi _2}\Gamma \int \limits _{\Omega _e}\Upsilon _i \Upsilon _j d\Omega _e,\\&-\Gamma (1-\Gamma )\int \limits _{\Omega _e}\Upsilon _i \frac{d\Upsilon _j}{d\Gamma }d\Omega _e,\ L^{23}_{ij} = 2\lambda \Gamma \int \limits _{\Omega _e}\Upsilon _i \Upsilon _j d\Omega _e,\ L^{31}_{ij}=L^{34}_{ij}=L^{35}_{ij} =L^{36}_{ij} =0,\ L^{32}_{ij}=-2\lambda \Gamma \int \limits _{\Omega _e}\Upsilon _i \Upsilon _jd\Omega _e,\\ L^{33}_{ij}= & {} -\frac{1}{\chi _1\chi _2}\int \limits _{\Omega _e}\frac{d\Upsilon _i}{d\eta }\frac{d\Upsilon _j}{d\eta }d\Omega _e +\frac{1}{2}(1-\Gamma )\eta \int \limits _{\Omega _e}\Upsilon _i \frac{d\Upsilon _j}{d\eta }d\Omega _e+\Gamma \int \limits _{\Omega _e} \bar{F_1}\Upsilon _i\frac{d\Upsilon _j}{d\eta }d\Omega _e -\Gamma \int \limits _{\Omega _e}\bar{H}\Upsilon _i \Upsilon _j d\Omega _e\\&-\Gamma (1-\Gamma )\int \limits _{\Omega _e}\Upsilon _i \frac{d\Upsilon _j}{d\Gamma }d\Omega _e, L^{41}_{ij} = L^{42}_{ij} = L^{43}_{ij} = 0,\\ L^{44}_{ij}= & {} -\frac{\chi _3}{\chi _4}\int \limits _{\Omega _e} \frac{d\Upsilon _i}{d\eta } \frac{d\Upsilon _j}{d\eta }d\Omega _e + \frac{Pr}{2}(1-\Gamma )\eta \int \limits _{\Omega _e}\Upsilon _i \frac{d\Upsilon _j}{d\eta }d\Omega _e +P_r\zeta \int \limits _{\Omega _e} \bar{F_1}\Upsilon _i\frac{d\Upsilon _j}{d\eta }d\Omega _e +P_rN_b\int \limits _{\Omega _e}\bar{\Phi }'\Upsilon _i \frac{d\Upsilon _j}{d\eta }d\Omega _e\\&+P_rN_t\int \limits _{\Omega _e}\bar{\Theta }'\Upsilon _i \frac{d\Upsilon _j}{d\eta }d\Omega _e -P_r\Gamma (1-\Gamma )\int \limits _{\Omega _e}\Upsilon _i \frac{d\Upsilon _j}{d\Gamma }d\Omega _e, L^{45}_{ij}=L^{46}_{ij}=L^{51}_{ij} =L^{52}_{ij}=L^{53}_{ij}= L^{56}_{ij} = 0,\\ L^{54}_{ij}= & {} -\frac{N_t}{N_b}\int \limits _{\Omega _e}\frac{d\Upsilon _i}{d\eta }\frac{d\Upsilon _j}{d\eta }d\Omega _e, L^{55}_{ij}= -\int \limits _{\Omega _e} \frac{d\Upsilon _i}{d\eta } \frac{d\Upsilon _j}{d\eta }d\Omega _e + \frac{S_c}{2}(1-\Gamma )\eta \int \limits _{\Omega _e}\Upsilon _i \frac{d\Upsilon _j}{d\eta }d\Omega _e +S_c\Gamma \int \limits _{\Omega _e} \bar{F_1}\Upsilon _i\frac{d\Upsilon _j}{d\eta }d\Omega _e\\&- S_c\Gamma (1-\Gamma )\int \limits _{\Omega _e}\Upsilon _i \frac{d\Upsilon _j}{d\Gamma }d\Omega _e, L^{61}_{ij}= L^{62}_{ij} = L^{63}_{ij} = L^{64}_{ij} = 0,\\ L^{65}_{ij}= & {} -P_e\delta _1\int \limits _{\Omega _e} \frac{d\Upsilon _i}{d\eta } \frac{d\Upsilon _j}{d\eta }d\Omega _e, L^{66}_{ij}= -\int \limits _{\Omega _e} \frac{d\Upsilon _i}{d\eta } \frac{d\Upsilon _j}{d\eta }d\Omega _e + \frac{S_b}{2}(1-\Gamma )\eta \int \limits _{\Omega _e}\Upsilon _i \frac{d\Upsilon _j}{d\eta }d\Omega _e +S_b\Gamma \int \limits _{\Omega _e} \bar{F_1}\Upsilon _i\frac{d\Upsilon _j}{d\eta }d\Omega _e\\&-P_e\int \limits _{\Omega _e}\bar{\Phi }'\Upsilon _i\frac{d\Upsilon _j}{d\eta }d\Omega _e - P_e\int \limits _{\Omega _e}\bar{\Phi }'' \Upsilon _i{d\Upsilon _j}d\Omega _e - S_b\Gamma (1-\Gamma )\int \limits _{\Omega _e}\Upsilon _i \frac{d\Upsilon _j}{d\zeta }d\Omega _e, \end{aligned}$$

and

38 $$\begin{aligned} R^1_i= & {} 0, \ R^2_i = -\oint \limits _{\Gamma _e} \Upsilon _i n_{\eta }\frac{\partial {H}}{\partial \eta } \,ds, \ R^3_i = -\oint \limits _{\Gamma _e} \Upsilon _i n_{\eta }\frac{\partial {F_2}}{\partial \eta } \,ds, \ R^4_i = -\oint \limits _{\Gamma _e} \Upsilon _i n_{\eta }\frac{\partial \Theta }{\partial \eta }\,ds,\nonumber \\ R^5_i= & {} - \oint \limits _{\Gamma _e} \Upsilon _i n_{\eta }\frac{\partial \Phi }{\partial \eta }\,ds - \frac{N_t}{N_b}\oint \limits _{\Gamma _e}\Upsilon _i n_{\eta }\frac{\partial \Theta }{\partial \eta } \,ds,\ R^6_i = - \oint \limits _{\Gamma _e} \Upsilon _i n_{\eta }\frac{\partial \chi }{\partial \eta }\,ds. \end{aligned}$$

where, $\bar{F}_1 = \sum _{j=1}^4 \bar{F}_{1j} \Upsilon _j$, $\bar{H} = \sum _{j=1}^4 \bar{H}_j \Upsilon _j$, $\bar{F}_2 = \sum _{j=1}^4 \bar{F}_{2j}\Upsilon _j$, $\bar{\Theta }' = \sum _{j=1}^4 \bar{\Theta }'_j\Upsilon _j$, and $\bar{\Phi }'= \sum _{j=1}^4 \bar{\Phi }'_j\Upsilon _j$ supposed to be the known values. Compute 6 functions at each node. The obtained system of equations 61,206 are nonlinear after assembly, linearize using an iterative algorithm with the $10^{-5}$ precision necessary.

## Results and discussion

We have demonstrated the importance of nanoparticle aggregation on the dynamics of suspensions containing microscopic particles spinning fluid susceptible to Lorentz and Coriolis forces, as well as gyrotactic microorganisms in this section. In every one of the figures, set of two curves are drawn for two specific cases: (1) $\Phi _{int} = 1.0$ (non-aggregated nanoparticles) and (2) $\Phi _{int} \ne 1.0$ (aggregated nanoparticles). Further, the default values for other involved parameters and quantities are: $P_r = 6.2$ (water-host fluid), $M = 1.0$, $N_b = 0.2, N_t = 0.2, lambda =1.0$, $S_c = 10.0$, $S_b = 5.0$, $P_e = 0.5$, $D = 1.8$, $\delta _1 = 0.2$, $\Phi = 0.01$, $\Phi _{max} = 0.650$, and $R_a/R_p = 3.34$. To verify the reliability and validity of Galerkin finite element approach, a grid independence study is performed. The problem input is distributed into various mesh density, and there is no more fluctuation is noted after $100\times 100$, so we draw all the results on $100\times 100$ grid size (see Table 3). To show that the current results are validate and reliable, a comparison with recently published studies are presented in Tables 3 and 4 in specific cases. The present outcomes are very close with the already published results, as evidenced. The friction factors along with primary and secondary directions $-F_1''(0) \& -F_2(0)$ in Table 4 against growing inputs of $\lambda = 0.0, 1.0, 2.0, 5.0$ at $\Gamma = 1.0$. The results achieved are in excellent agreement with those anlyzed by Ali et al.^45^, and Wang^17^. Additionaly, in Table 5, the $-\Theta (0)$ inputs are acknowledged between Adnan et al.^52^ and Bagh et al.^53^, and present FEM results against growing inputs of $\lambda \& M$, and discovered that they are in accord. As a result, the numerical computations may be validated, and the Finite Element Computations produced using Matlab program have a high convergence rate.

**Figure 3:**
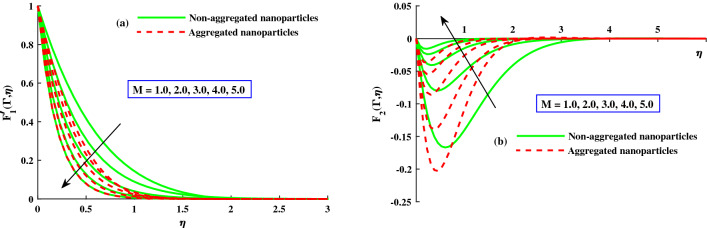
Variation of *M* on $F_{1}^{'}(\Gamma ,\eta )$ in axial, and $F_{2}^{'}(\Gamma ,\eta )$ in transverse.

**Figure 4:**
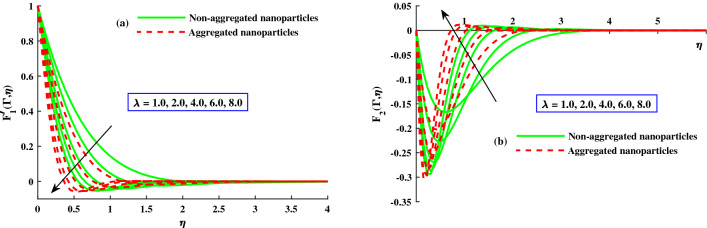
Variation of $\lambda$ on $F_{1}^{'}(\Gamma ,\eta )$ in axial, and $F_{2}^{'}(\Gamma ,\eta )$ in transverse.

**Figure 5:**
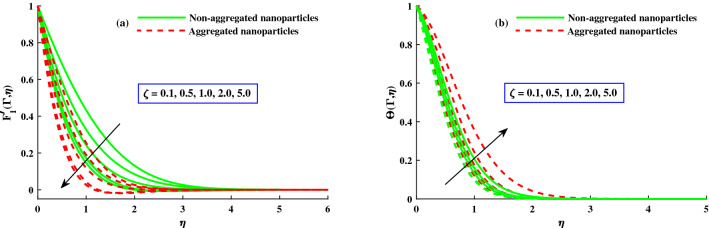
Variation of $\zeta$ on $F_{1}^{'}(\Gamma ,\eta )$ in x-direction, and $\Theta (\Gamma ,\eta )$.

The distribution of primary velocity $F_1'(\Gamma ,\eta )$ and secondary velocity $F_2'(\Gamma ,\eta )$ against exceeding inputs of magnetic (*M*) and rotating $(\lambda )$ parameters are depicted in Figs. 3 and 4 respectively. Figure 3a,b portraits the $F_1'(\Gamma ,\eta )$ and $F_2(\Gamma ,\eta )$ for distinct inputs of magnetic field. The enhanced magnetic field caused to produce the resistive force which called it Lorentz force and goes to recede of the primary velocity in Fig. 3b, whereas an inverse action is reported for secondary velocity in Fig. 3b. The impact of rotation parameter $\lambda$ on axial velocity $F_1'(\Gamma ,\eta )$ and transverse velocity $F_2(\Gamma ,\eta )$ portrayed in Fig. 4a,b. It is observed that diminishing of axial velocity for exceeding inputs of *lambda* because of Coriolis force while an opposing action is claimed for transverse velocity in Fig. 4b. The role of $\zeta$ (unsteady parameter) on axial velocity and thermal profile is deliberated in Fig. 5a,b. The proceeding inputs of $\zeta$ the axial velocity curve reduced while thermal distribution improved. Hence, it clear that the time dependent parameter is play significance role in controlling the momentum and thermal boundary thickness. Further, from these figures, the model along with nanoparticles aggregation has a lower distribution of primary velocity $F_1'(\Gamma ,\eta )$ and magnitude of secondary velocity $F_2'(\Gamma ,\eta )$, whereas distribution of primary and secondary velocities are slightly greater than that considering the model of homogeneous (non-aggregated nanoparticles). Physically, the formation of nanoparticles aggregation caused to increase in the effective viscosity^54^, and growing strength of viscosity is responsible to slow down the fluid velocity^55^.

**Figure 6:**
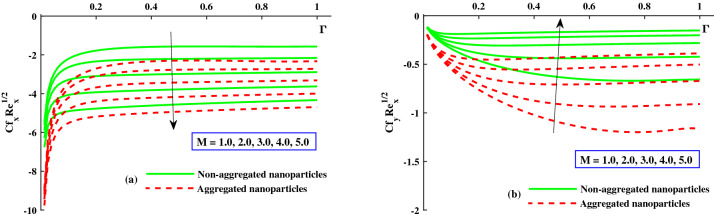
Variation of *M* on $Cf_xRe^{1/2}_x$ in x-direction, and $Cf_yRe^{1/2}_y$ in y-direction.

**Figure 7:**
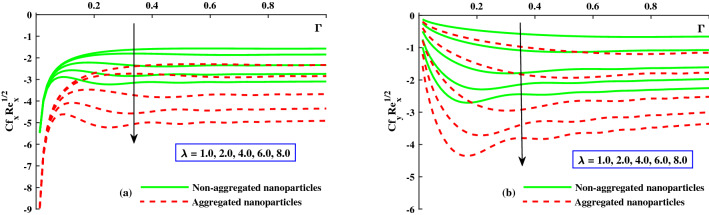
Variation of $\lambda$ on $Cf_xRe^{1/2}_x$ in x-direction, and $Cf_yRe^{1/2}_y$ in y-direction.

The distribution of friction factors $C_{f_x}{Re_x}^{1/2}$ (axial direction) and $C_{f_y}{Re_x}^{1/2}$ (transverse direction) against exceeding values of $\Gamma (0:0.2:1)$ and *M*(1 : 1 : 5) parameters are depicted in Fig. 6a,b. Figure 6a demonstrates that for growing $\Gamma (0\rightarrow 1$, the axial friction factor $(C_{f_x}{Re_x}^{1/2})$ is enhanced steadily rise to a fixed rate, after which no noticeable change is noticed, but for increasing *M*, a remarkable diminution in axial friction factor $(C_{f_x}{Re_x}^{1/2})$ is observed. For increasing $\Gamma (0\rightarrow 1$, the transverse direction friction factor $(C_{f_y}{Re_x}^{1/2})$ magnitude is steadily lowered until it reaches a constant rate, after which no appreciable difference is noticed, as illustrated in Fig. 6b, while improving *M*, and see the significance difference near the surface. Figure 7a,b depicts that for growing $\Gamma (0\rightarrow 1$, the axial skin friction $(C_{f_x}{Re_x}^{1/2})$ is progressively increased until it reaches a constant rate, afterwards which no substantial change is detected, whereas raising $\lambda$ requires a large drop in axial direction skin factor $(C_{f_x}{Re_x}^{1/2})$ and transverse direction $(C_{f_y}{Re_x}^{1/2})$ is noticed. Furthermore, it is apparent from these graphs that the ranges of $(C_{f_x}{Re_x}^{1/2})$ and $(C_{f_y}{Re_x}^{1/2})$ for the model along with nanoparticles aggregation has a negatively lower distribution as compared to non-aggregated nanoparticles case.

**Figure 8:**
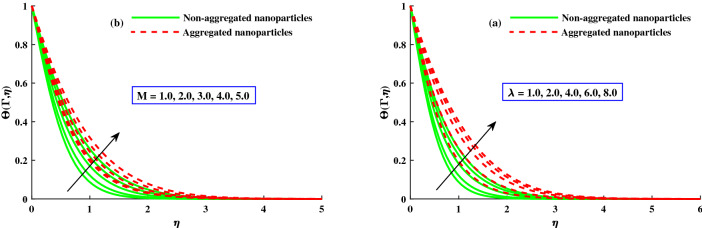
Variation of *M* and $\lambda$ on $\Theta (\Gamma ,\eta )$.

**Figure 9:**
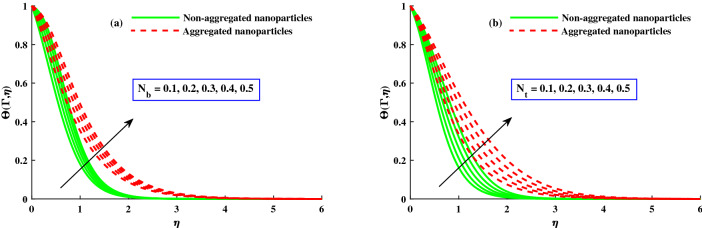
Variation of $N_b$ and $N_t$ on $\Theta (\Gamma ,\eta )$.

**Figure 10:**
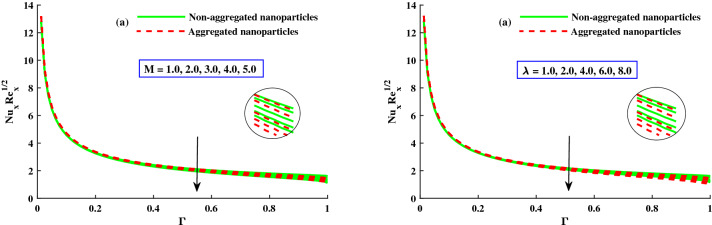
Variation of $Nu_xRe_x^1/2$ against *M*, and $\lambda$.

The distribution of $\Theta (\Gamma ,\eta )$ for different parameters is displayed in Figs. 8 and 9. The magnetic field parameter upgraded the $\Theta (\Gamma ,\eta )$ (temperature distribution) which clearly seen in Fig. 8a. It is because of net force mentioned as Lorentz force around the internal electric force and external magnetic field control the temperature profile, which is showed in Fig. 8a, while the thermal boundary layer thickness is improved against increasinng $\lambda$ as depict in Fig. 8b. Figure 9a,b displays that $\Theta (\Gamma ,\eta )$ for distict inputs of thermophoresis $(N_t)$ and Brownian motion $(N_b)$ parameters. The exceeding strength of $N_t \& N_b$ caused to increased the distribution of temperature profile. The higher $N_b$, the quicker the erratic movement of nano particles in the flow domain, the better the thermal dispersion. Furthermore, the thermophorestic (*Nt*) effect drives micro entities to move from a hotter to a cooler location, boosting the $\Theta (\Gamma ,\eta )$. Further, from these figures, the model without nanoparticles aggregation (homogeneous model) has a lower distribution of temperature $\Theta (\Gamma ,\eta )$, whereas distribution of $\Theta (\Gamma ,\eta )$ is slightly greater than that considering the model of nanoparticles aggregation. This result show that the nanoparticles aggregation has a positive effect on the nanofluid thermal conductivity^56,57^. The sketches of local Nusselt number $(Nu_{x}{Re_x}^{1/2})$ is depicted in Fig. 10a,b for $M(1:1:5) \& \lambda (1:2:8)$. For growing $M \& \lambda$, the distribution of $(Nu_{x}{Re_x}^{1/2})$ is decreased gradually. The nanoparticles aggregation model show a significant reduction in $(Nu_{x}{Re_x}^{1/2})$, whereas distribution of $Nu_{x}{Re_x}^{1/2}$ is slightly greater than that non-aggregated nanoparticles case.

**Figure 11:**
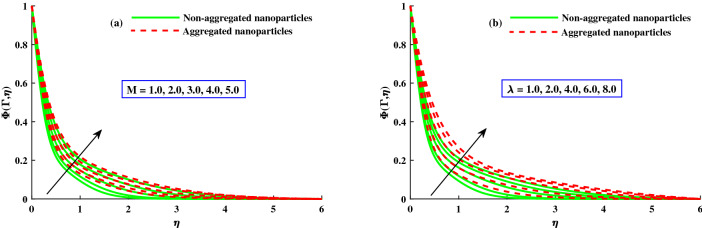
Variation of *M* and $\lambda$ on $\Phi (\Gamma ,\eta )$.

**Figure 12:**
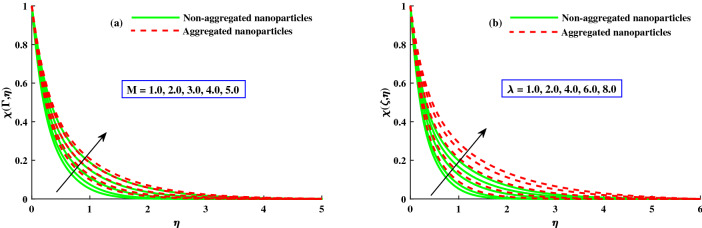
Variation of *M* and $\lambda$ on $\chi (\Gamma ,\eta )$.

**Figure 13:**
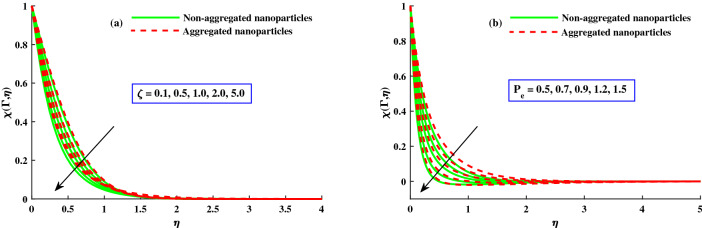
variation of *Lb* and *Pe* on $\chi (\Gamma ,\eta )$ at $\zeta = 1$.

**Figure 14:**
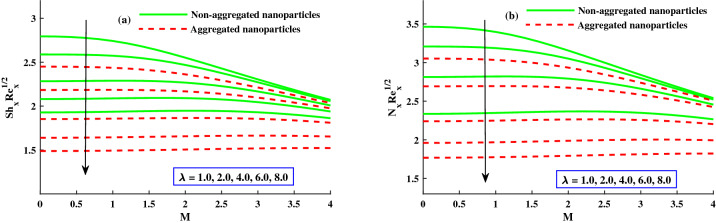
Variation of $Shr_xRe_x^1/2$ against $N_b$, $N_t$, *M*, and $\lambda$.

The distribution of nanoparticles volume fraction $\Phi (\Gamma ,\eta )$ and motile micoorganisms $\chi (\Gamma ,\eta )$ against exceeding inputs of magnetic (*M*) and rotating $(\lambda )$ parameters are depicted in Figs. 11 and 14 respectively. The tiny particles ($\Phi (\Gamma ,\eta )$) and motile microorganisms $(\chi (\Gamma ,\eta ))$ profiles are upgraded for growing strength of magnetic and rotatory parameters as portraits in Figs. 11a,b and 14a,b. For exceeding values of $\zeta$ (time-dependent parameter) and Peclet number $(P_e)$ parameters, the diminution of the thickness of the motile distribution is delineated in Figs. 12a,b. Hence, it clear that the time dependent parameter is play significance role in controlling the motile boundary thickness. Further, from these figures, the model along with nanoparticles aggregation has a greater distribution of concentration distributions, whereas distribution of nanoparticles and motile microorganisms primary are slightly greater than that considering the model of homogeneous (non-aggregated nanoparticles). The behavior of local Sherwood number $(Shr_{x}{Re_x}^{1/2})$ and motile microorganism density number $Re_x^{1/}N_x$ is deliberated in Fig. 13a,b for enhancing strength of $M(0{:}1{:}4) \& \lambda (1{:}2{:}8)$, respectively. For enhancing $M \& \lambda$, the distribution of motile microorganism density number $Re_x^{1/}N_x$ and $(Shr_{x}{Re_x}^{1/2})$ is declined. and it is also witnessed that the non-aggregated case has larger $Shr_{x}{Re_x}^{1/2}$ and $Re_x^{1/}N_x$ than that of aggregated case (Fig. 14).

## Conclusions

In this work, the Galerkin finite element study on the dynamics of rotating water based silver tiny particles subject to Coriolis, and Lorentz forces has been explored numerically along with swimming of motile organisms. The effective nanofluid viscosity and thermal conductivity has been studied by the authors for applying nanoparticles aggregation and homogeneous models. Depending on the outcomes of the analysis, it is reasonable to conclude that:

1. Exceeding values in the strength of Coriolis and Lorentz has a receding impact on the axial momentum and transverse momentum magnitude, and
  an enhancing influence on the profiles of thermal and concentrations boundary layers.
  Enhance the magnitude of $Cf_xRe_x^{1/2}$ (skin friction factor).
  a negative effects on $Nu_{x}{Re_x}^{1/2}$, $Shr_{x}{Re_x}^{1/2}$, and $N_{x}{Re_x}^{1/2}$.
  A similar trend against higher values of rotation is reported by Oke et al.^21^, and found that the increasing rotation caused to enhance the magnitude of skin friction coefficient, and mean while magnetic caused to decline in $Nu_{x}{Re_x}^{1/2}$.
2. Growing strength of Brownian motion, thermophoresis, and time-dependent parameters have an enhancing effect on the thermal distribution. The higher Bronian motion, the quicker the movement of nano particles in the flow domain, the better the thermal dispersion, and the thermophorestic effect drives micro entities to move from a hotter to a cooler location which caused to boosting the temperature^23,35^.
3. Motile microorganism concentration diminishes against incremented Peclet number and time-dependent values.
4. Formation of nanoparticles aggregation has a declining impact on the axial and transverse velocities magnitude, but
  an exceeding impact on the profiles of temperature, tiny particles volume fraction, and motile microorganism.
  the nanoparticles aggregation case has lower the values of $C_{f_x}{Re_x}^{1/2}$ and $C_{f_y}{Re_x}^{1/2}$.
  the nanoparticles aggregation model show a significant reduction in $Nu_{x}{Re_x}^{1/2}$.
  the non-aggregated case has larger $Shr_{x}{Re_x}^{1/2}$ and $Re_x^{1/}N_x$ than that of aggregated case.

This work can be extended in the future for non-Newtonian based fluids susceptible to nanoparticles and other physical characteristics after a victorious simulated strife of parametric effects on fluid dynamics

**Table 3:** Analysis of grid independence for distinct grid sizes at $\zeta = 1.0$.

Grid size	$-F_1''(\zeta ,0)$	$-F_2'(\zeta ,0)$	$-\Theta '(\zeta ,0)$	$-\Phi '(\zeta ,0)$	$-\chi '(\zeta ,0)$
20 $\times$ 20	2.2314	1.2404	0.4194	2.0326	2.7752
30 $\times$ 30	2.2172	1.2294	0.4367	1.9184	2.7376
50 $\times$ 50	2.2129	1.2168	0.4462	1.8603	2.6754
80 $\times$ 80	2.2122	1.2109	0.4463	1.8479	2.6461
100 $\times$ 100	2.2119	1.2094	0.4456	1.8451	2.6389
120 $\times$ 120	2.2118	1.2090	0.4454	1.8448	2.6386

**Table 4:** Comparative of skin friction $-F_{1}^{'}(0)$ and $-F_{2}^{''}(0)$ for different inputs of $\lambda$ at $zeta=1$ while other factors are ignored.

$\lambda$	Ali et al.^45^		Wang.^17^		Present	
	$-F_{1}^{''}(0)$	$-F_{2}^{'}(0)$	$-F_{1}^{''}(0)$	$-F_{2}^{'}(0)$	$-F_{1}^{''}(0)$	$-F_{2}^{'}(0)$
0.0	1.00000	0.00000	1.0000	0.0000	1.00000	0.00000
1.0	1.32501	0.83715	1.3250	0.8371	1.32501	0.83715
2.0	1.65232	1.28732	1.6523	1.2873	1.65232	1.28732
5.0	2.39026	2.15024	–	–	2.39026	2.15024

**Table 5:** Comparative of $-\theta '(0)$ for different inputs of $\lambda$ at $\xi =1$ when others physical involved parameters are negligible.

$\lambda$	Adnan et al.^52^	Bagh et al.^53^	FEM (current outcomes)	
	$M = 0.0, Pr = 2.0$	$M = Pr = 2.0$	$M = 0.0, Pr = 2.0$	$M = Pr = 2.0$
0.0	0.911	0.6682	0.91107	0.66821
0.5	0.853	0.6627	0.85343	0.66268
1.0	0.770	0.6483	0.77028	0.64828
2.0	0.638	0.6030	0.63805	0.60303

## Data Availability

The data used to support the findings of this study are available from the corresponding author upon request.
